# Improved prediction of MHC-peptide binding using protein language models

**DOI:** 10.3389/fbinf.2023.1207380

**Published:** 2023-08-17

**Authors:** Nasser Hashemi, Boran Hao, Mikhail Ignatov, Ioannis Ch. Paschalidis, Pirooz Vakili, Sandor Vajda, Dima Kozakov

**Affiliations:** ^1^ Division of Systems Engineering, Boston University, Boston, MA, United States; ^2^ Department of Electrical and Computer Engineering, Boston University, Boston, MA, United States; ^3^ Department of Applied Mathematics and Statistics, Stony Brook University, Stony Brook, NY, United States; ^4^ Laufer Center for Physical and Quantitative Biology, Stony Brook University, Stony Brook, NY, United States; ^5^ Department of Biomedical Engineering, Boston University, Boston, MA, United States; ^6^ Department of Chemistry, Boston University, Boston, MA, United States

**Keywords:** MHC class I, deep learning, transformers, natural language processing, cellular immune system

## Abstract

Major histocompatibility complex Class I (MHC-I) molecules bind to peptides derived from intracellular antigens and present them on the surface of cells, allowing the immune system (T cells) to detect them. Elucidating the process of this presentation is essential for regulation and potential manipulation of the cellular immune system. Predicting whether a given peptide binds to an MHC molecule is an important step in the above process and has motivated the introduction of many computational approaches to address this problem. NetMHCPan, a pan-specific model for predicting binding of peptides to any MHC molecule, is one of the most widely used methods which focuses on solving this binary classification problem using shallow neural networks. The recent successful results of Deep Learning (DL) methods, especially Natural Language Processing (NLP-based) pretrained models in various applications, including protein structure determination, motivated us to explore their use in this problem. Specifically, we consider the application of deep learning models pretrained on large datasets of protein sequences to predict MHC Class I-peptide binding. Using the standard performance metrics in this area, and the same training and test sets, we show that our models outperform NetMHCpan4.1, currently considered as the-state-of-the-art.

## 1 Introduction

Major Histocompatibility Complex molecules (MHC) are large cell surface proteins that play a key role in immune response by detecting and responding to foreign proteins and antigens. An MHC molecule detects and binds to a peptide (a small fragment of a protein derived from an antigen), creating a peptide-MHC complex, and presents it to the surface of the cell; then, based on the interactions between this complex and the T cell receptor at the cell surface, an immune response is triggered to control the compromised cell (Maimela et al., 2019; Janeway et al., 2001; Teraguchi et al., 2020; Ong et al., 2021). MHC molecules are classified into two classes: (i) MHC Class I which controls non-self intracellular antigens by presenting antigenic peptides (of 8–14 sequence length) to cytotoxic T cell lymphocytes (CD8^+^ TCR) and (ii) MHC Class II, which controls extracellular antigens by presenting antigenic peptides (of 13–25 sequence length) to helper T cell lymphocytes (CD4^+^ TCR). One of the main steps in studying the role of the MHC molecules in the immune system is developing insights into the interactions of the MHC molecules and non-self pathogen peptides, referred to as MHC-peptide binding (Reynisson et al., 2020). MHC-peptide binding prediction plays an important role in vaccine design and studies of infectious diseases, autoimmunity, and cancer therapy (O’Donnell et al., 2020; Grebenkin et al., 2020).

There are two basic experimental methods to study MHC-peptide binding: (i) Peptide-MHC binding affinity (BA) assays in which, given a peptide, binding preferences of different MHC molecules to the peptide are measured (Townsend et al., 1990); (ii) MHC associated eluted ligands (EL) generated by Liquid Chromatography Mass Spectrometry (LC-MS) in which, based on a single experiment, a large number of eluted ligands corresponding to an MHC are identified (Caron et al., 2015). Compared to the BA method, the EL method is highly accurate and thorough and it is a reliable way to determine the peptides included in the immunopeptidome (namely, the entire set of peptides forming MHC-peptides complexes (Alvarez et al., 2019)). Both methods, however, are labor-intensive and time-consuming. As a result, a number of computational methods have been developed to predict MHC-peptide binding (Boehm et al., 2019). These methods include heuristic approaches using MHC allele–specific motifs to identify potential ligands in a protein sequence (Bui et al., 2005), supervised machine learning approaches, including artificial neural networks (ANN) (Nielsen et al., 2003), hidden Markov models (HMM) (Zhang et al., 2006), and regression models (Parker et al., 1994; Doytchinova and Flower, 2001). The performance of these machine learning methods increases with the amount of data available in epitope databases such as SysteMHC (Shao et al., 2018) and Immune Epitope Database (IEDB) (Vita et al., 2019). While some of these methods are trained for only one specific MHC allele (known as allele-specific methods), there are more generalized models (pan-specific methods) where a single model covers all of alleles of interest. The methods are also categorized by the type of predicted variables. Among these methods, some have been shown to be more promising, such as NetMHCpan (Reynisson et al., 2020), DeepLigand (Zeng and Gifford, 2019), and MHCflurry (O’Donnell et al., 2020; Aranha et al., 2020). The most recent version of NetMHCpan (NetMHCpan 4.1) is currently considered as the state-of-the-art in the MHC Class I-peptide binding prediction problem (Reynisson et al., 2020).

NetMHCpan is a pan-specific model which predicts binding of peptides to any MHC molecule of known sequence using artificial neural networks. Since 2003, this model has gradually been improved and its last version for MHC Class I (NetMHCpan 4.1) has been introduced in 2020. This model is trained on a combination of the BA and EL peptide datasets where the inputs are sequences associated with MHC-peptide complexes (Tong, 2013). There are some specific features associated with this method that helps it to outperform other approaches: (i) instead of using the complete sequence of MHC molecules as input, NetMHCpan uses pseudo-sequences of MHC molecules with a fixed length (34 amino acids); these pseudo-sequences include those amino acids associated with the binding sites of the MHC molecule inferred from *a priori* knowledge; (ii) to accommodate peptides of different lengths (8–15 in MHC Class I), the length is fixed to a uniform length of 9 *via* insertion and deletion of amino acids; (iii) additional features with specificity information of the peptides are used during the insertion and deletion steps; for example, the original length of the peptide is encoded as a categorical variable and the length of the sequence that was inserted/deleted is added as a different feature; (iv) NetMHCpan consists of several shallow neural networks and it implements the ensemble technique: using cross-validation, the training dataset is split into 5 parts and the model is trained five times, one for each split. Also, NetMHCpan uses shallow neural networks with one hidden layer containing 56 or 66 neurons that are trained using 10 different random initial weight configurations; thus, the ensemble NetMHCpan contains 100 different models.

As indicated above, the most recent NetMHCpan approach [version 4.1 (Reynisson et al., 2020)] is based on shallow neural networks. In recent years, a number of more complex yet efficient methods such as deep neural networks have shown promising results in a number of fields (Deng et al., 2013; LeCun et al., 2015; Khan and Yairi, 2018; Voulodimos et al., 2018; Iuchi et al., 2021; Mohammadzadeh and Lejeune, 2021). For example, transformer models (Vaswani et al., 2017), a recent breakthrough in natural language processing, have shown that large models trained on unlabeled data are able to learn powerful representations of natural languages and can lead to significant improvements in many language modeling tasks (Devlin et al., 2018; Hu et al., 2022). Furthermore, it has been shown that collections of protein sequences can be treated as sentences so that similar techniques can be used to extract useful biological information from protein sequence databases (Rao et al., 2019; Rives et al., 2019). A highly successful example of this approach has been DeepMind’s recent protein-folding method, using attention-based models (Jumper et al., 2020; Lensink et al., 2021; Egbert et al., 2021; Ghani et al., 2021). Currently, there are a number of publicly available pre-trained models which have been shown to be helpful in a variety of downstream protein related tasks (Rao et al., 2019; Rives et al., 2019; Elnaggar et al., 2020; Rao et al., 2020; Rao et al., 2021).

In the work reported in this paper, we consider using a number of such pre-trained models and Deep Learning (DL) methods to address the MHC Class I peptide binding prediction problem. One component of the approach in this work is based on transfer learning. In Deep Learning (DL), transfer learning is a method in which a DL model is first trained on a problem similar to the problem of interest; then, a portion or the whole of this pre-trained model is used for training the model of the desired problem. This approach is particularly advantageous when the amount of data for the problem of interest is limited, however, large databases associated with other problems with some similarity to the problem of interest exist (Fu and Bates, 2022). Fine-tuning a pre-trained model using the dataset associated with the problem of interest is one of the approaches in transfer learning and one that is used in this work. In this case, a portion, or all of the weights associated with the pre-trained model are used as the initial weights of a new DL model for the desired task. For example, in NLP, BERT (Bidirectional Encoder Representations from Transformers) is a pre-trained transformer model which is trained on a large corpus of unlabelled text including the entire Wikipedia (about 2,500 million words) and the Book Corpus (800 million words) (Devlin et al., 2018). Thereafter, the pre-trained model has been used for a number of NLP tasks such as text classification, text annotation, question answering, and language inference, to name a few.

Recently, following the successful results of pre-trained transformer models such as BERT and their transfer learning derivatives in NLP applications, similar approaches have been attempted in the protein field thanks to the substantial growth in the number of protein sequences. As a result, there are a number of pre-trained self-supervised BERT-like models applied to protein data in the form of unlabeled amino acid sequences which can be very useful for many protein task-specific problems using transfer learning (Elnaggar et al., 2020; Rao et al., 2020). Two recent works have considered using protein language models in the MHC-peptide binding problem. BERTMHC (Cheng et al., 2020) explores whether pre-trained protein sequence models can be helpful for MHC Class II-peptide binding prediction by focusing on algorithms that predict the likelihood of presentation of a peptide given a set of MHC Class II molecules. They show that models generated from transfer learning can achieve better performance on both binding and presentation prediction tasks compared to NetMHCIIpan4.0 (last version of NetMHCpan in MHC Class II (Reynisson et al., 2020)). Another BERT-based model known as ImmunoBERT (Gasser et al., 2021) applies pre-trained transformer models in the MHC Class I-peptide binding problem. As reported, in this work they were not able to compare their model fairly with NetMHCPan (Reynisson et al., 2020) and MHCflurry (O’Donnell et al., 2020) performance due to a lack of access to the same training set. BERTMHC and ImmunoBERT both use the TAPE pre-trained models (Rao et al., 2019) which were trained on 31 million protein sequences, whereas now there are larger and more informative pre-trained models available such as ESM (Rao et al., 2020; Lin et al., 2022) and ProtTrans (Elnaggar et al., 2020) which are trained on more than 250 million protein sequences.

In the work reported in this paper, we focus on MHC Class I-peptide binding prediction and develop different approaches using the larger pre-trained protein language models. Two of these approaches are based on fine-tuning using a soft-max layer in one and a Graph Attention Network (GAT) in the other. Our third approach is based on a domain adaptation method to further pre-train the protein language models and enhance the fine-tuning performance. We evaluate the performance of our models using the standard metrics of the field and the same training and test sets as those of NetMHCpan 4.1. We show that our methods outperform NetMHCpan 4.1 over these test sets.

## 2 Materials and methods

### 2.1 Methods

**FIGURE 1:**
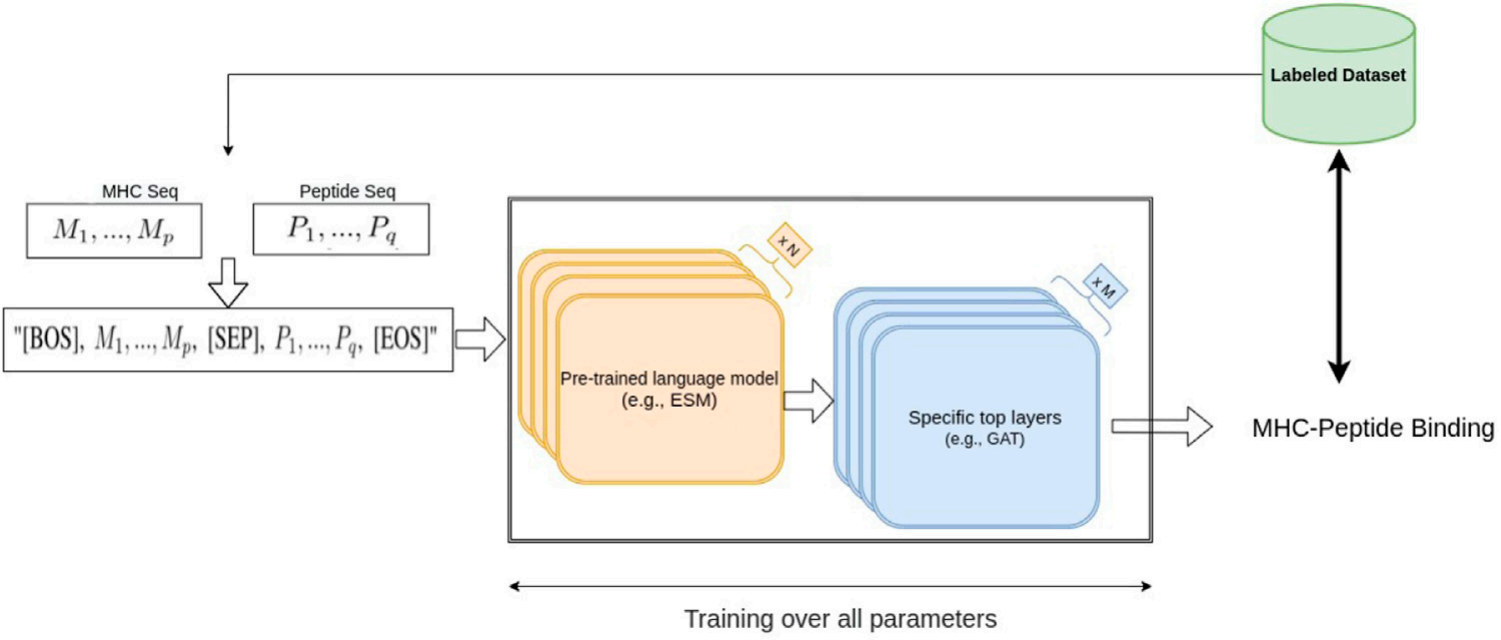
Our fine-tuning architecture using NLP-based pre-trained models.

In this work, we considered two large protein language pre-trained models, ESM1b (Rao et al., 2020) and ESM2 (Lin et al., 2022), two BERT-based models which are trained on hundreds of millions of protein sequences. ESM1b is a pre-trained Transformer protein language model from Facebook AI Research which has been shown to outperform all tested single-sequence protein language models across a range of protein structure prediction tasks (Rao et al., 2020); its successor, ESM2, has achieved even better performance on protein folding related tasks. ESM1b and ESM2-650M have 33 layers with 650 million parameters and an embedding dimension of 1280, and the largest model we used, ESM2-3B, has 36 layers, embedding dimension of 2560 and 3 billion parameters. In our fine-tuning approaches, after including an additional layer at the end of the ESM models, we re-trained the entire set of parameters of ESM1b and ESM2 and trained the parameters of the added layer using the MHC-peptide dataset. Thus, the entire parameters, including the pre-trained weights of the model, were updated based on our dataset (Figure 1).

#### 2.1.1 ESM fine-tuning

Since ESM models can be regarded as transformer-based bidirectional language models (bi-LM), we borrowed an idea from a basic NLP task called Natural Language Inference (NLI) (Bowman et al., 2015) to perform MHC-peptide binding prediction. One of the NLI tasks is the sequence-pair classification problem, namely, predicting whether a text A (e.g., “rabbits are herbivorous”) can imply the semantics in a text B (e.g., “rabbits do not eat rats”). Similarly, in the MHC-peptide case, we would like to know whether a given peptide sequence (same as text A) binds to a given MHC sequence (same as text B), suggesting that applying an NLI-based model could be effective in MHC-peptide binding prediction. A common transformer-based NLI model combines text A and B into one sequence “[BOS] seq-A [SEP] seq-B [EOS]” as input, where [BOS], [SEP] and [EOS] are special tokens^1^ in bi-LM vocabulary.

Suppose the amino acids in the MHC and the peptide sequences are *M*_1_, …, *M*_*p*_ and *P*_1_, …, *P*_*q*_, respectively. We generate the sequence “[BOS], *M*_1_, …, *M*_*p*_, [SEP], *P*_1_, …, *P*_*q*_, [EOS]” with length *p* + *q* + 3 as the ESM model input, and obtain the same size embedding vectors $v_{\mathrm{BOS}},v_{M_{1}},\ldots ,v_{M_{p}},v_{\mathrm{SEP}},v_{P_{1}},\ldots ,v_{P_{q}},v_{\mathrm{EOS}}$ from the last layer of ESM models, corresponding to the special tokens and the amino acids in the MHC and the peptide. As a common strategy in NLP sequence classification tasks, we use the embedding of [BOS] to be the MHC-peptide sequence-pair embedding vector $\bar{v}$ (Ibtehaz and Kihara, 2023). Finally, passing $\bar{v}$ through a softmax classifier layer, we output the probability of binding and use it to compute the loss and apply back-propagation. Compared to embedding the MHC and the peptide separately, this compound input allows the transformer to use the attention mechanism to further extract the interactive information between the amino acids in the MHC and the peptide, thus, helping the binding prediction.

Although ESM models are well pre-trained in an unsupervised manner, using a large number of universal sequences, we know that MHCs are highly specific types of protein sequences, so the embedding from the pre-trained ESM models may not be optimal for the specific MHC task and input format. Therefore, we not only need to train the final softmax classifier but need to train the ESM model parameters as well to improve the sequence-pair embedding. We applied a fine-tuning which is commonly used in NLP. Initialized from the pre-trained ESM model parameters, we updated the parameters in the whole network using a small learning rate during the back-propagation, so that valuable information in the pre-trained ESM models is maintained while the fine-tuned ESM models provided a more informative embedding specific to the MHC tasks.

#### 2.1.2 ESM domain adaptation

In NLP, domain adaptation pre-training is an important tool to introduce domain-specific information into a bi-LM. A BERT model pre-trained on general corpora (Devlin et al., 2018) (e.g., Wikipedia) can be further pre-trained by the same masked language modeling (MLM) methods but using corpora from specific domains such as clinical medicine (Alsentzer et al., 2019) in order to gain better down-stream task performance in different knowledge domains. This idea fits our protein language models as well because ESM models were pre-trained on general full protein sequences whereas our MHC-peptide binding prediction focuses on MHC pseudo-sequences and short peptides, which were not available in the ESM pre-training data. Therefore, we applied domain adaptation to the ESM models in order to offer the ESM models more knowledge about the MHC pseudo-sequences and the peptides.

We still use the NetMHCpan V4.1 training set as our domain adaptation pre-training set. For an MHC-peptide pair “[BOS], *M*_1_, …, *M*_*p*_, [SEP], *P*_1_, …, *P*_*q*_, [EOS]”, we first randomly mask 7 amino acids (around 15%), and then feed this masked sequence pair to the pre-trained ESM models. Note that with a probability of 0.8, an amino acid to be masked will be masked by a special token [MASK], otherwise it will be “masked” by the original amino acid, which resembles the MLM setting in BERT. The ESM models will then exploit the information from the visible context of amino acids, and finally use a classification head to predict the masked amino acids. As a result, the special structural characteristics of the MHC pseudo-sequences and the peptides will be further learned, and our domain-adapted ESM models can better fit the MHC-related tasks, compared with the vanilla ESM models. For one MHC-peptide pair, the loss to be minimized is the mean cross-entropy loss between the predicted and the ground truth masked amino acids. During the ESM domain adaptation pre-training, we still update all parameters of the ESM models, and our domain-adapted ESM models will be used as the initialization of the MHC-peptide binding prediction fine-tuning task described in the previous section.

#### 2.1.3 ESM-GAT fine-tuning

Here, we consider our second approach to fine-tuning. Molecular structure-based biological data such as proteins, can be modeled with graph structures in which amino-acids or atoms are considered as nodes, and contacts or bonds are considered as edges. It has been shown that Graph Neural Networks (GNNs), as a branch of deep learning in non-Euclidean spaces, perform well in various applications in bioinformatics (Zhang et al., 2021). In our context, the interaction between an MHC and a peptide can be described by a graph in which the amino-acids are considered as the nodes and the interaction between them as edges. To model such a graph information, we added a variant model of GNN known as Graph Attention Network (GAT) as the last layer of the ESM network. GAT is a novel neural network architecture that operates on graph-structured data by leveraging attention layers to address the shortcomings of prior methods based on graph convolutions or their approximations (Veličković et al., 2017). For each MHC-peptide pair, we used a directed graph G , where the nodes *N*_1_, …, *N*_*p*+*q*+3_ represent the *p* + *q* + 3 amino acids and the special tokens as described above, and an edge (*N*_*i*_, *N*_*j*_) indicates that amino acids *i* and *j* are in contact with each other. Denote the neighbor set of amino acid *i* as $A\left(i\right)=\left\{j:\left(N_{i},N_{j}\right)\in G\right\}$ ; then, each embedding vector **v**_*i*_ is updated as a weighted average of its transformed neighbor embedding vectors:

$$v_{i}^{\prime }=\underset{j\in A\left(i\right)}{\sum }\alpha_{ij}Wv_{j},$$

where **W** is a weight matrix for vector transformation, and the weight *α*_*ij*_ is computed using an attention mechanism. Suppose **z**_*ij*_ is the concatenation of vectors **Wv**_*i*_ and **Wv**_*j*_ and **c** is a parameter vector, then the weight *α*_*ij*_ is given by:

$$\alpha_{ij}=\frac{\exp\left(\sigma \left(\langle {c,z}_{ij}\rangle \right)\right)}{\sum_{k\in A\left(i\right)}\exp\left(\sigma \left(\langle {c,z}_{ik}\rangle \right)\right)},$$

where *σ* is an activation function. Note that the attention mechanism here is known as *additive* attention, which is different from the dot-product attention mechanism used in ESM and other transformer-based models.

After each GAT layer, we update the embedding vector for the amino acids and the special tokens as $v_{\mathrm{BOS}}^{\prime },v_{M_{1}}^{\prime },\ldots ,v_{M_{p}}^{\prime },v_{\mathrm{SEP}}^{\prime },v_{P_{1}}^{\prime },\ldots ,v_{P_{q}}^{\prime },v_{\mathrm{EOS}}^{\prime }$ , and more GAT layers follow. Here, in our implementation, we use two fully connected GAT layers. Same as vanilla transformer model (Vaswani et al., 2017), we apply multi-head attention mechanism in which for each GAT layer, we split the parameters and pass each split independently through a separate head. Particularly, in the first GAT layer we use 8 attention heads which are then concatenated together and passed to the next layer while in the final GAT layer we average the heads of a certain token. We finally use the embedding vector of [BOS] in the final GAT layer as the MHC-peptide sequence pair embedding vector to determine the binding prediction. The final GAT layer was meant to use the attention mechanism to aggregate all the node information into [BOS] position by letting [BOS] token contact with all the amino acids in the graphs which makes the [BOS] embedding potentially a more powerful sequence embedding than simply using the average of the embedding vectors output by the first GAT layer. Compared to using only ESM dot-product attention layers and a linear classification head, now we are adding more GAT additive attention layers to dynamically refine the ESM embedding and enhance the final binding classification.

Note that the contact information can be defined differently through graphs. If in the absence of specific information about the contacts, fully-connected graphs are used as we did, the dependency among any amino acids can be further exploited by those additive attention layers, similar to the ESM layers. However, if prior information on contacts is available and is represented in the graphs, such information can also be introduced to the GAT layers by allowing the additive attention mechanism to happen only between the desired amino acids.

### 2.2 Dataset

#### 2.2.1 Training set

We used the training set used by the last version of NetMHCpan (Reynisson et al., 2020), including 13 millions binary labeled MHC-peptide binding samples, generated from two main data sources: (i) the BA peptides derived from in-vitro Peptide-MHC binding assays, and (ii) the EL peptides derived from mass spectrometry experiments. However, it has been shown that the results from the mass spectrometry EL experiments are mostly poly-specific, i.e., they contain ligands matching multiple binding motifs (Alvarez et al., 2019). That being said, for most of the samples in the EL dataset, each peptide is associated with multiple alleles (from 2 to 6 alleles for each peptide). Thus, in this training set, the EL dataset is composed of two subsets: (i): Single-Allele (SA) peptides assigned to single MHCs and (ii) Multi-Allele (MA) peptides with multiple MHC options to be assigned. Table 1 shows the distribution of the aforementioned dataset which indicates that more than 67% of the dataset is associated with EL-MA. According to (Alvarez et al., 2019), the existence of the MA dataset introduces some challenges in terms of data analysis and interpretation; therefore, to train a binary MHC-peptide predictor, a process, known as deconvoluting the MA binding motifs, is needed to convert these EL-MA data to a single peptide-MHC pair (Reynisson et al., 2020).

**TABLE 1:** Distribution of training set used in NetMHCpan 4.1 (Reynisson et al., 2020); Columns correspond to each type of training data, for which the number of positive and negative samples, and the total amount of unique MHCs are shown. A threshold of 500 nM is used to define positive BA data points.

Binding affinity			EL (single allele)			EL (multi allele)		
Positives	Negatives	MHCs	Positives	Negatives	MHCs	Positives	Negatives	MHCs
52,402	155,691	170	218,962	3,813,877	142	446,530	8,395,021	112

#### 2.2.2 Deconvolution of multi allelic (MA) data

To deconvolute the EL-MA dataset, several computational approaches have been used based on unsupervised sequence clustering (Bassani-Sternberg and Gfeller, 2016; Bassani-Sternberg et al., 2017). Although these methods show some progress in dealing with the MA dataset, they have some shortcomings; for example, they do not work in cell lines including MHC alleles with similar binding motifs. Therefore, in the new version of NetMHCPan (Version 4.1), they present a new framework, NNAlign-MA (Alvarez et al., 2019), which works better than the previous approaches. NNAlign-MA is a neural network framework, which is able to deconvolute the MA dataset during the training of the MHC-peptide binding predictor. Recently (Cheng et al., 2020), attempted to solve this problem in MHC Class II by using a multiple instance learning (MIL) framework. MIL is a supervised machine learning approach, where the task is to learn from data including positive and negative bags of instances. Each bag may contain many instances and a bag is labeled positive if at least one instance in it is positive (Maron and Lozano-Pérez, 1998). Assume the *i*-th bag includes m alleles as *A*_*i*_ = {*a*_*i*1_, *a*_*i*2_, …, *a*_*im*_} which is associated with peptide sequence *s*_*i*_. At each training epoch, for each instance in the *i*-th bag, *x*_*ij*_ = (*a*_*ij*_, *s*_*i*_), the probability of whether that instance is positive, *p*(*y*_*ij*_ = 1|*x*_*ij*_) is defined as ${\hat{y}}_{ij}=f_{\theta }\left(a_{ij},s_{i}\right)$ where *f*_*θ*_ is the neural network model; in (Cheng et al., 2020) max pooling is used as a symmetric pooling operator to calculate the prediction of the bag from the predictions of instances within it. Here, in our work, we follow this MIL idea to deal with the EL-MA dataset.

#### 2.2.3 Test set

In order to have a fair comparison of our model and NetMHCPan 4.1, we used the same test set as provided in their work (available in the Supplementary Section). This dataset is associated with a collection of 36 EL-SA datasets, downloaded from (Abelin et al., 2017). Each dataset is well enriched, length-wise, with a number of negative decoy peptides equal to 5 times the number of ligands of the most abundant peptide length.

### 2.3 Metric

Predicting the binding affinity of MHC with a peptide is a binary classification problem. Typical metrics for assessing the quality of binary classification models for a given task include precision, accuracy, recall, receiver operating characteristic curve (ROC) and the corresponding Area Under the Curve (AUC). In this work, we use AUC-ROC and a specific precision metric known as positive predictive value (PPV); AUC and PPV have been used as the main metrics in previous works in MHC-peptide binding prediction (Reynisson et al., 2020; O’Donnell et al., 2020). AUC is an evaluation metric for binary classification problems which measures the area under the ROC curve. AUC ranges in value from 0 to 1 and models with higher AUC perform better at distinguishing between the positive and negative classes. PPV is another metric which specifically is defined in this area and is interpretable as a model’s ability to rank positive samples far above the negative samples. PPV is defined as fraction of true positive samples (hits) among the top-scoring $\frac{1}{N+1}$ fraction of samples, assuming that the ratio of the number of positive samples to negatives (decoys) is 1: N (known as hit-decoy ratio). Since NetMHCpan (Reynisson et al., 2020) uses hit-ratio 1:19 and MHCflurry (O’Donnell et al., 2020) uses hit-ratio 1:99, here in this work, we use both.

Beyond AUC-ROC and PPV, we also consider three more metrics: F1 score, Precision-Recall Area Under Curve (AUC-PR), and Matthews Correlation Coefficient (MMC). These metrics provide a comprehensive evaluation of the model’s performance by measuring the balance between precision and recall, and summarizing performance on imbalanced datasets.

## 3 Results

In order to evaluate and compare the performance of our approaches with the state-of-the-art method, we used the latest version of NetMHCpan server (Version 4.1); as mentioned above, the same training and test sets from (Reynisson et al., 2020) were used in this study. The list of independent EL SA test set including the MHC molecules, the number of peptides and the distribution of positives and negatives for each case is provided in the Supplementary Material.

**FIGURE 2:**
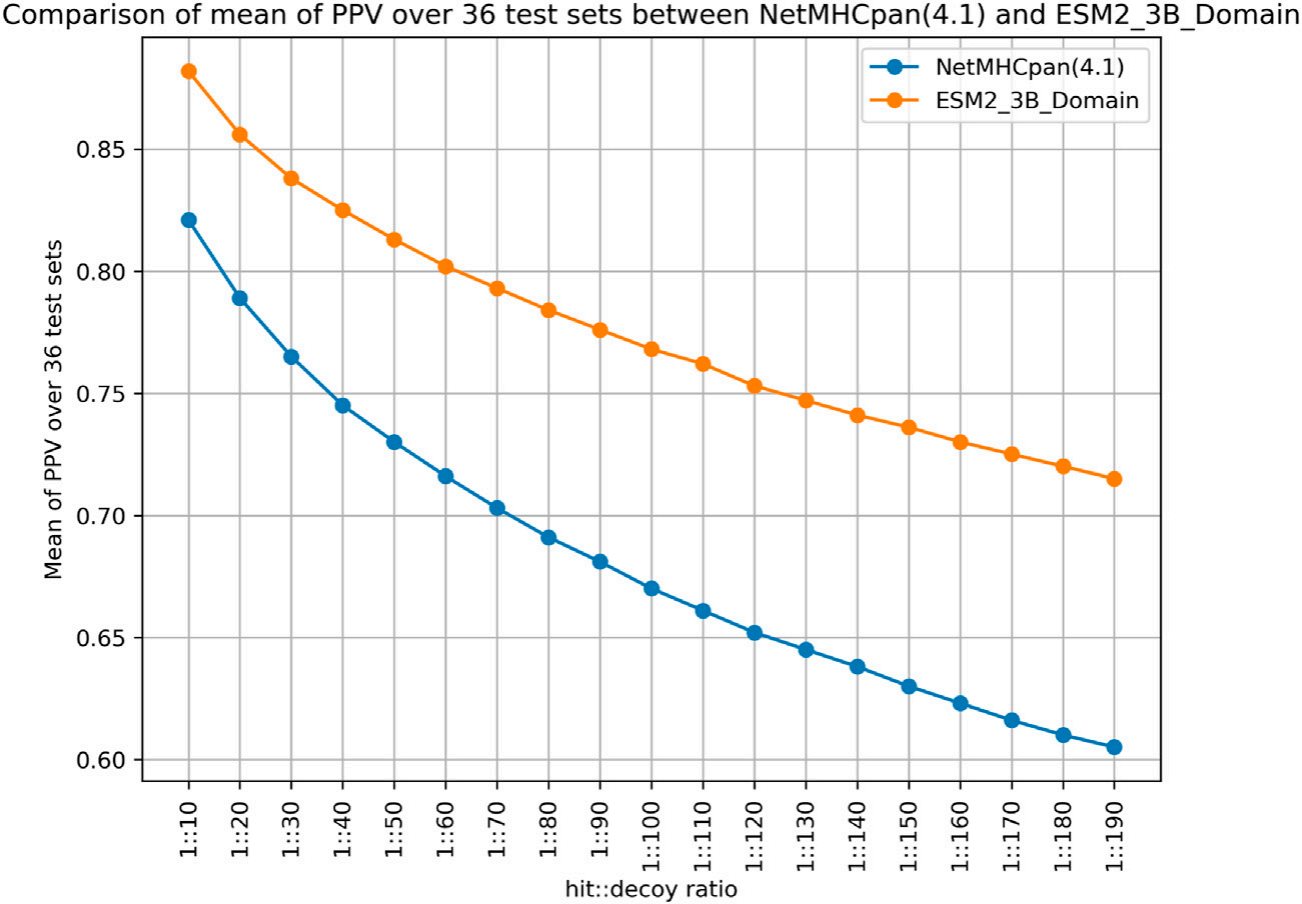
PPV Comparison of ESM2 model vs NetMHCpan 4.1 over different hit-decoy ratio.

To arrive at the hit-decoy ratios of 1:19 and 1:99 for each case, we followed a random sampling approach that was repeated 1000 times. As a result, for each MHC molecule, the PPV values are sample averages of 1000 values. Additionally, in Figure 2 we provide a comparison over a range of hit-decoy ratios.

To present the results of the comparison of our fine-tuning as well as our domain adaptation approaches with NetMHCpan, we provide two figures for each hit-decoy ratio in what follows: (a) a bar plot that provides a comparison of PPVs of our approach and NetMHCpan for each MHC molecule in the test set, and (b) a scatter plot of the same PPV values that provides a better visual summary of performance comparison.

Since there was not a significant difference in performance when using the ESM1b, ESM2-650M, or ESM2-3B, we report the ESM2-3B values in this section which were slightly better in mean performance than others. Tables 2, 3 below show the summary of the results for fine-tuning and domain adaptation which provides the mean of using PPV, AUC-ROC, F1, AUC-PR, and MMC averages over all MHC molecules in the test set.

**TABLE 2:** Summary table of comparison of the mean of our models and NetMHCpan (V4.1) AUC-ROC and PPV over all test sets.

Models	PPV (hit-decoy ratio: 1:19)	PPV (hit-decoy ratio: 1:99)	AUC-ROC
NetMHCpan4_ 1	0.791	0.671	0.950
ESM1b	0.834	0.737	0.977
ESM2_650M	0.837	0.742	0.976
ESM2_3B	0.844	0.753	0.976
ESM1b_domain	0.851	0.756	0.979
ESM2_650M_domain	0.850	0.756	0.980
ESM2_3B_domain	0.857	0.769	0.981

**TABLE 3:** Summary table of comparison of the mean of our models and NetMHCpan (V4.1) F1, AUC-PR, and MMC over all test sets.

Models	Fl	AUC-PR	MMC
NetMHCpan4_l	0.711	0.833	0.726
ESM1b	0.771	0.885	0.779
ESM2 650M	0.785	0.888	0.791
ESM2 3B	0.788	0.893	0.794
ESM1b_domain	0.794	0.902	0.799
ESM2_650M_domain	0.796	0.900	0.801
ESM2 3B domain	0.801	0.908	0.806

### 3.1 ESM fine-tuning

**FIGURE 3:**
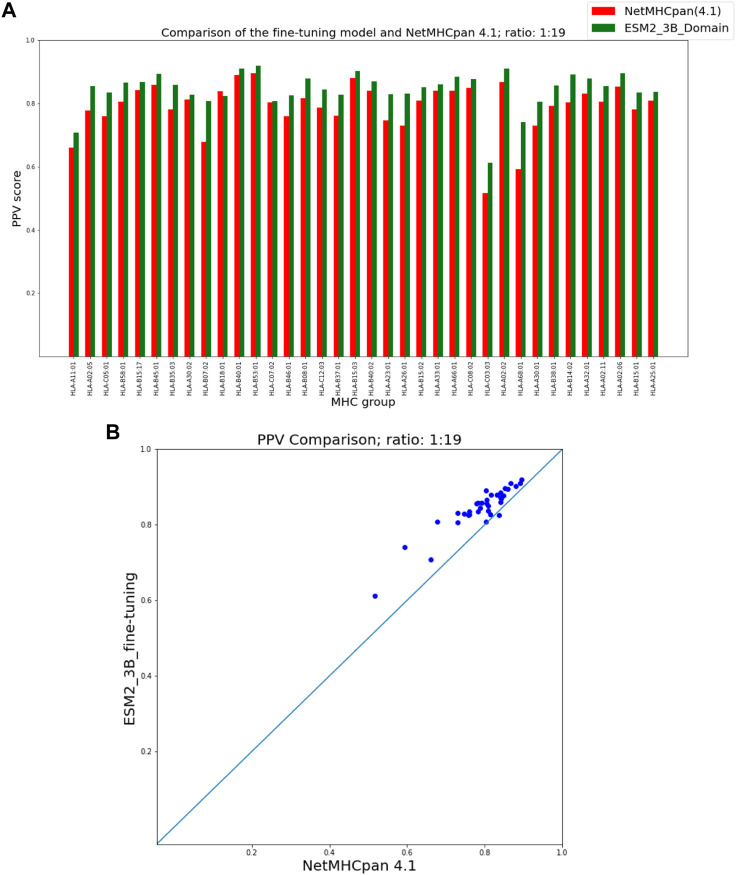
PPV Comparison (hit-decoy ratio: 1:19) of our fine-tuning method with the latest NetMHCpan server (Version 4.1) over the same training and test sets (Reynisson et al., 2020). **(A)** Bar plots associated with each test set. **(B)** Scatter plot: each point is the PPV of each group of test set.

**FIGURE 4:**
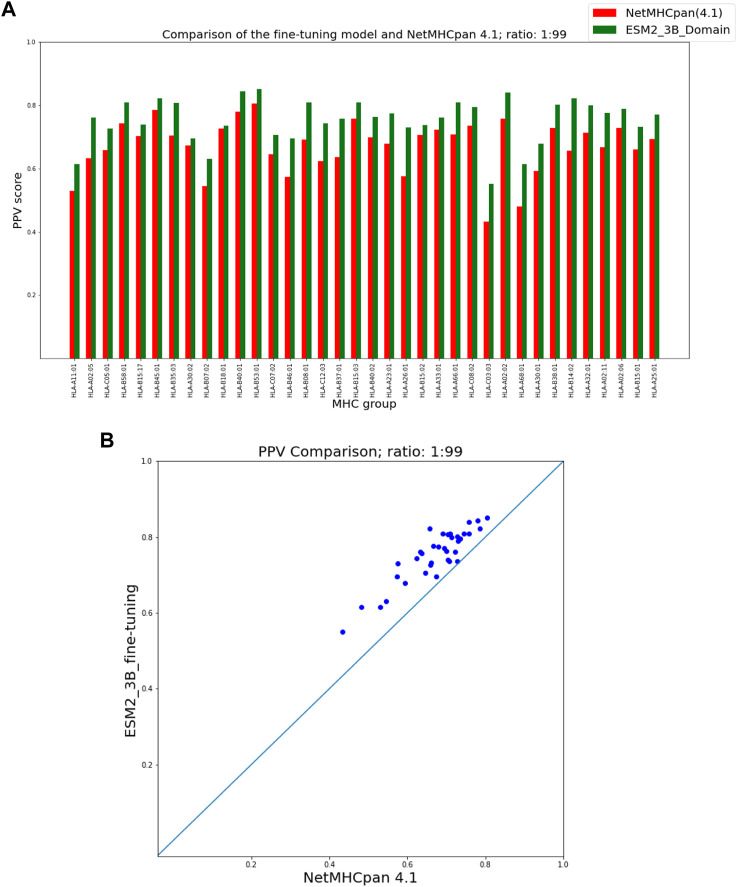
PPV Comparison (hit-decoy ratio: 1:99) of our fine-tuning method with the latest NetMHCpan server (Version 4.1) over the same training and test sets (Reynisson et al., 2020). **(A)** Bar plots associated with each test set. **(B)** Scatter plot: each point is the PPV of each group of test set.

As seen in Figure 3 our fine-tuning method outperforms NetMHCpan over all hit-decoy ratios in the 35 different test sets; only for HL-B18:01, at ratio 1:19, NetMHCpan performs slightly better. Also, as seen in Figure 4, at ratio 1:99 the model outperforms NetMHCpan for all 36 test set including the HL-B18:01.

### 3.2 ESM domain adaptation

**FIGURE 5:**
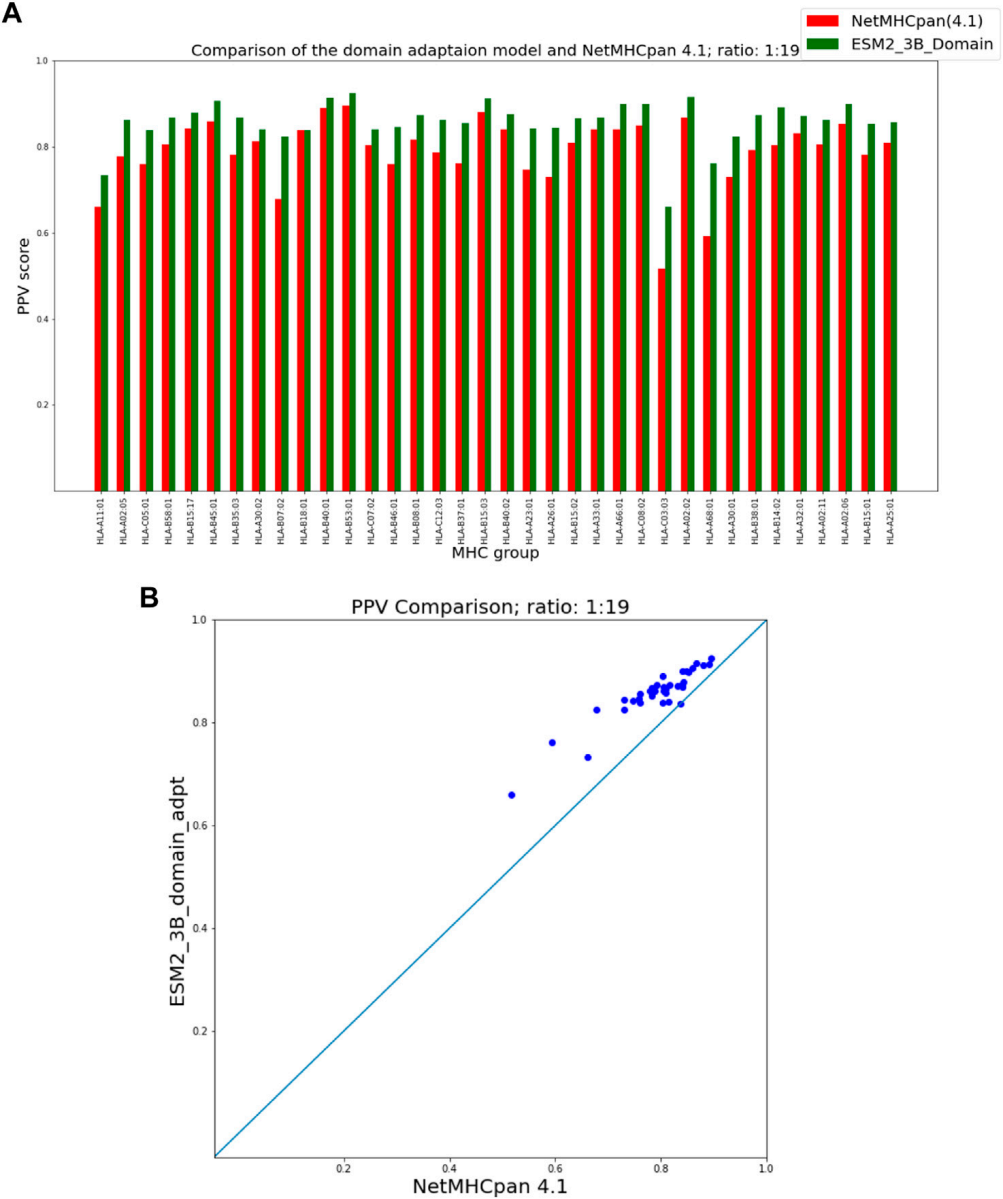
PPV Comparison (hit-decoy ratio: 1:19) of our domain-adaptation method with the latest NetMHCpan server (Version 4.1) over the same training and test sets (Reynisson et al., 2020). **(A)** Bar plots associated with each test set. **(B)** Scatter plot: each point is the PPV of each group of test set.

**FIGURE 6:**
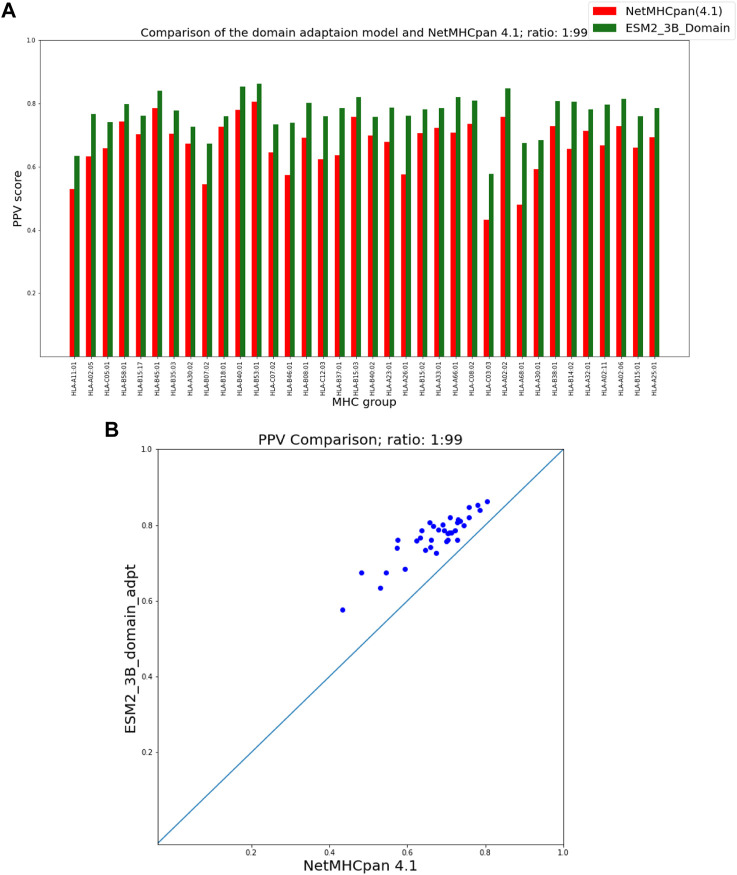
PPV Comparison (hit-decoy ratio: 1:99) of our domain-adaptation method with the latest NetMHCpan server (Version 4.1) over the same training and test sets (Reynisson et al., 2020). **(A)** Bar plots associated with each test set. **(B)** Scatter plot: each point is the PPV of each group of test set.

Figures 5, 6 show that our domain adaptation model outperforms NetMHCpan over all hit-decoy ratios in the 35 different test sets; only for HL-B18:01, at ratio 1:19, NetMHCpan slightly performs better. In addition, the performance of the domain adaptation approach is slightly better than the fine-tuning approach.

### 3.3 ESM-GAT fine-tuning

**FIGURE 7:**
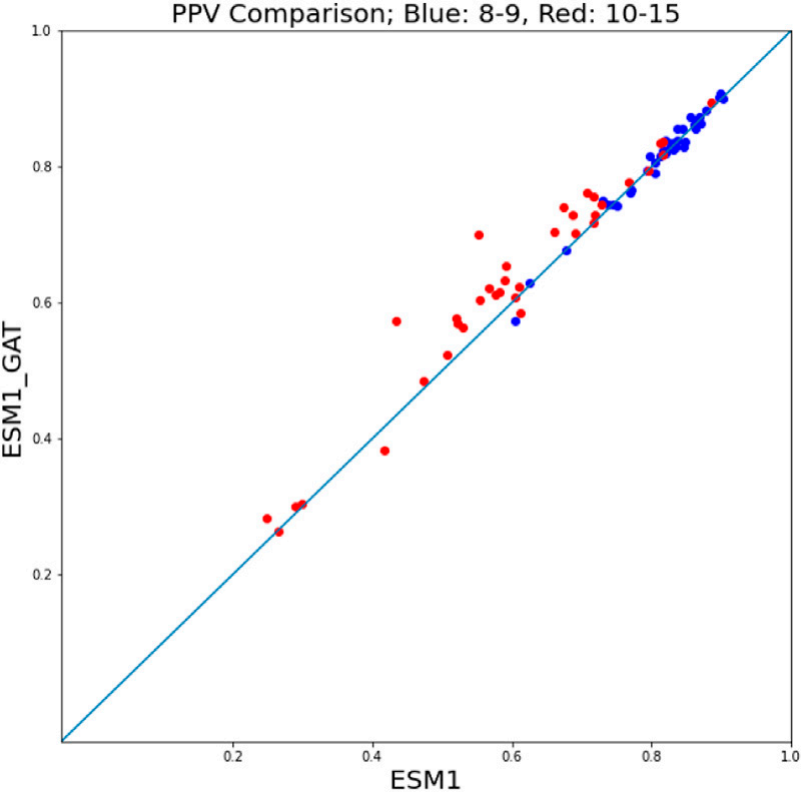
ESM-GAT fine-tuning outperforms the ESM fine-tuning method when the test set with peptide length 10–15 is considered (red points) while the results are almost the same when using the test set with peptides of length 8 and 9 (blue points).

Given the superior performance of ESM fine-tuning in comparison with NetMHCpan, to assess the performance of ESM-GAT fine-tuning, we compared its performance with that of ESM fine-tuning. In this case, a hit-decoy ratios of 1:19 was considered. We found that in the case where we subdivided the training and test sets between peptides of length 8 and 9 on the one hand and peptides of size 10–15 on the other, ESM-GAT fine-tuning outperformed ESM fine-tuning. Specifically, we used subsets of the training set that included samples associated with peptides of length 8 and 9 and compared both methods over two test sets. As can be seen in Figure 7, ESM-GAT outperformed ESM fine-tuning when the test set with peptide length 10–15 was considered (red dots), while the results were almost the same when using the test set with peptides of length 8 and 9 (blue dots). Bar plots associated with these figures are available in the Supplementary Section. This observation suggests that GAT has the potential to improve the ability of the model to predict binding of peptides with lengths different from those considered in the training set. The testing of this conjecture is a subject of future research.

## 4 Conclusion

Predicting peptides that bind to the major histocompatibility complex (MHC) Class I is an important problem in studying the immune system response and a plethora of approaches have been developed to tackle this problem. NetMHCpan 4.1 is developed based on training shallow neural networks (Reynisson et al., 2020) and is currently considered the state-of-the-art for MHC Class I-peptide binding prediction. A number of recent works have focused on using protein language models in MHC-peptide binding problems. Protein language models developed based on deep learning approaches, such as attention-based transformer models, have shown significant progress towards solving a number of challenging problems in biology, most importantly, the protein structure prediction problem (Jumper et al., 2021). BERTMHC (Cheng et al., 2020) and ImmunoBERT (Gasser et al., 2021) for the first time applied the pre-trained protein language models in MHC-peptide binding problems. Both methods used a relatively small pre-trained model ((Rao et al., 2019) was trained with 31 million protein sequences); currently, there are substantially larger and more informative models such as ESM1b (Rao et al., 2020) and ProtTrans (Elnaggar et al., 2020) which are trained on more than 250 million protein sequences. In the work reported in this paper we focus on MHC Class I peptide binding prediction by developing approaches based on large pre-trained protein language models, ESM1b (Rao et al., 2020) and ESM2 (Lin et al., 2022). We follow two fine-tuning approaches using a soft-max layer and Graph Attention Network (GAT) as well as implement a domain adaptation pre-training for ESM models. In order to have a fair comparison, we train our model using the same training set used by NetMHCpan 4.1 (Reynisson et al., 2020) and evaluate our model using the same test set. We show, using the standard performance metrics in this area, that our model outperforms NetMHCpan. As reported in the paper, adding Graph Attention Network (GAT) to the ESM networks, improved the ability of the model to predict peptides with lengths different from those considered in the training set; this feature is expected to be beneficial for training models beyond MHC Type I.

## Data Availability

Publicly available datasets were analyzed in this study. This data can be found here: https://services.healthtech.dtu.dk/suppl/immunology/NAR_NetMHCpan_NetMHCIIpan/.

## Footnotes

1 A token is a string of contiguous characters between two spaces, or between a space and punctuation marks.
