# Supplementary material for: Improved prediction of MHC-peptide binding using protein language models
Source: Front Bioinform. 2023 Aug 17;3:1207380. doi: 10.3389/fbinf.2023.1207380 (PMC10469926; doi:10.3389/fbinf.2023.1207380)
Supplement: Supplementary file 1 [file Table1.pdf]

Supplementary Material

1 SUPPLEMENTARY TABLES AND FIGURES

1.1 Figures

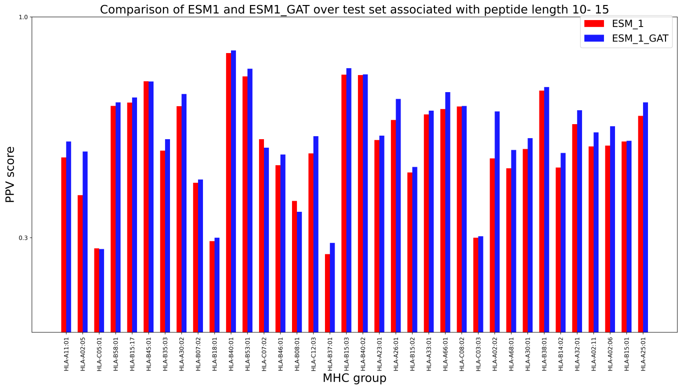

Figure S1. PPV Comparison (hit-decoy ratio: 19) of ESM fine-tuning method versus ESM-GAT over the training set with peptide length 8 and 9 and test set with peptide length 10 to 14.

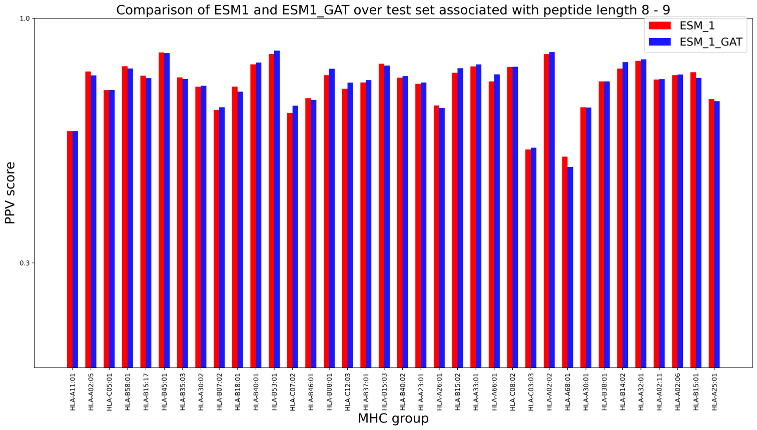

Figure S2. PPV Comparison (hit-decoy ratio: 19) of ESM fine-tuning method versus ESM-GAT over the training set with peptide length 8 and 9 and test set with peptide length 8 and 9..

Table S1. Independent EL SA test set provided by NetMHCpan 4.1

MHC	# Peptides	# Positives	# Negatives
HLA-A02:02	77053	3063	73990
HLA-A02:05	45136	2016	43120
HLA-A02:06	54510	1975	52535
HLA-A02:11	48445	2035	46410
HLA-A11:01	33424	2309	31115
HLA-A23:01	30467	1697	28770
HLA-A25:01	6906	396	6510
HLA-A26:01	7730	555	7175
HLA-A30:01	15837	892	14945
HLA-A30:02	33180	2415	30765
HLA-A32:01	28036	1436	26600
HLA-A33:01	43333	2138	41195
HLA-A66:01	41538	1988	39550
HLA-A68:01	5648	433	5215
HLA-B07:02	2469	159	2310
HLA-B08:01	3365	180	3185
HLA-B14:02	21601	1056	20545
HLA-B15:01	16624	769	15855
HLA-B15:02	16702	637	16065
HLA-B15:03	44968	1953	43015
HLA-B15:17	45917	1712	44205
HLA-B18:01	18284	784	17500
HLA-B35:03	8275	330	7945
HLA-B37:01	20048	1253	18795
HLA-B38:01	9509	619	8890
HLA-B40:01	18908	1268	17640
HLA-B40:02	23768	1333	22435
HLA-B45:01	18750	760	17990
HLA-B46:01	14015	575	13440
HLA-B53:01	46991	2016	44975
HLA-B58:01	17946	866	17080
HLA-C03:03	35568	2003	33565
HLA-C05:01	7033	383	6650
HLA-C07:02	15293	593	14700
HLA-C08:02	32416	1546	30870
HLA-C12:03	36448	1273	35175
